# Supplementary material for: Plasma membrane H+-ATPase regulation is required for auxin gradient formation preceding phototropic growth
Source: Mol Syst Biol. 2014 Sep 26;10(9):751. doi: 10.15252/msb.20145247 (PMC4299663; doi:10.15252/msb.20145247)
Supplement: Supplementary file 1 — Supplementary Information [file msb0010-0751-SD1.pdf]

Table of Contents

Supplementary Material and Methods

Supplementary Figure S1: Phototropic response of various *aux1lax* mutants and expression of *AUX1*, *LAX1*, *LAX2* and *LAX3* in etiolated hypocotyls.

Supplementary Figure S2: Impact of initial apoplastic pH on simulated auxin gradients.

Supplementary Figure S3: Impact of different irradiation directions on simulated auxin gradients.

Supplementary Figure S4: Phototropic response of *aha1-6* and *aha2-4* mutants.

Supplementary Figure S5: Hypocotyl elongation rate during phototropism.

Supplementary Figure S6: Analysis of auxin distribution in hypocotyls.

Supplementary Figure S7: Quantification of H^+-ATPase phosphorylation levels at the penultimate Thr.

Supplementary Figure S8: Phototropic response of the auxin biosynthesis mutants *sav3* and *yuc1yuc4*.

Supplementary Table S1: Parameters impacting auxin flux kinetics including their assumed values.

Supplementary Table S2: List of primers used in this study.

Supplementary information

Supplemental Material and Methods

RNA extraction and quantitative RT-PCR

50 Hypocotyls from 3 day-old etiolated seedlings were dissected using a dissection scissor under green safe light and immediately immersed into liquid N2. RNA extraction and quantitative real time PCR were performed as described in (Lorrain et al. 2009), except that 170 ng of RNA were used for the reverse transcription reaction. Primer efficiency was preliminary determined using a dilution serie of the cDNA preparation. Primers sequences are given in table S2. Data from three technical and three biological replicates were then analyzed and normalized with the value of the expression levels of *AUX1* of one of the replicate.

The model

To investigate lateral auxin gradient formation underlying phototropic bending in *Arabidopsis thaliana* hypocotyls, we constructed an auxin flux model that tracks auxin relocation as a function of different topological as well as biophysical parameters. The geometry of the model includes a transverse cross section through the hypocotyl explicitly featuring cellular compartments as well as apoplastic compartments. This cross section was determined using microscopy data and an overview on the modeled domain is given in Fig. 1*A*.

The model is based on a system of ordinary differential equations simulating the flux contributions between neighboring compartments. Free diffusion is assumed between the apoplast compartments, while we consider protonation state dependent auxin

diffusion and active auxin efflux based on carriers, i.e. those of the PIN and PGP families, on the interface between cellular compartments and the apoplastic space. We did not consider active IAA influx contributions resulting from AUX/LAX for the following reasons that are explained in the main manuscript.

Apart from the fluxes within the cross section, we explicitly allowed for exchange with the apoplastic space just above and below the considered cross section. Since gradient formation is assumed to happen locally (Iino 2001; Preuten et al. 2013), auxin concentrations in the apoplast just above and below are kept constant, thereby functioning as an auxin source or sink depending on auxin redistribution in the modeled cross section. Our simulations have shown that this exchange with surrounding cross sections has a cushioning effect on the strength of gradients predicted by our model: For example, setting the exchange between the modeled cross section and cross sections above and below to zero results in qualitatively similar results but stronger gradients (increasing gradient strength from 12% for vacuolated cells to 82%). And although this documents a considerable effect of coupling strength on gradient strength, the fact that the qualitative behavior in both extremes is similar lets us not further pursue this parameter.

In addition, we incorporate the fact that the stele is the major mode of basipetal auxin transport and therefore has to be considered an auxin source. Further incorporating the fact that auxin transport in the stele is faster than in the apoplast (Kramer 2006), for our model we assume that auxin concentrations in the stele are high and kept constant. The model thereby neglects a potential contribution of modulated auxin biosynthesis within cells of the modeled cross section. This assumption seems to be confirmed by phototropism assays in two auxin biosynthesis mutants that were previously shown to be defective for shade-induced auxin-dependent hypocotyl elongation *sav3* and

yuc1yuc4 (Tao et al. 2008; Won et al. 2011) but show normal phototropic responses (see Fig S8). Nevertheless, considering the redundancy in the *TAA1/TAR* and *YUC* gene families, a contribution of local auxin biosynthesis cannot be ruled out completely.

In this framework we define a lateral auxin gradient as follows: First, due to the type of manipulations we use in the simulations (e.g. side-directed pH manipulation, side-directed PIN localization changes) the modeled cross section can be divided in shaded and lit side just like for hypocotyls in experiments. Under this assumption, a gradient is defined as difference in intracellular auxin concentrations comparing epidermal cells on lit and shaded side by building ratios between the two. Whenever their ratio differs from '1' a gradient has formed. Its steepness is expressed in terms of percentage difference in auxin concentration between the two sides.

Model parameters

The model comprises three different types of parameters. First there is a set of kinetic constants that are inherently necessary to translate auxin fluxes into mathematical expressions. Although these constants ideally would be determined experimentally, many of them are difficult to measure and therefore have to be treated as free parameters (Kramer 2007). An overview on these parameters is given in Table S1.

All of the listed parameters apart from the decay rate μ had been part of a sensitivity analysis in which we varied each reported value separately by ±1 and ±2 orders of magnitude. During this analysis it turned that the dissociation constants K_{I} and K as well as the membrane permeability of membranes for auxin (P_{diff}) are rather negligible because varying them had no strong impact on the forming gradients. Instead, the model was sensitive to changes of the remaining three parameters, transport capacities of PIN carriers C_{I}, transport capacity of PGP carriers C, and

the diffusion constant of auxin D_{IAA}. It responded approximately linearly to changes of these parameters with pumping capacities increased by 2 orders of magnitude resulting in gradients of about 70% concentration difference between sides and a change of 2 orders of magnitude in the diffusion rate resulting in gradients increased by about 50% as compared to the reported diffusion constants.

The second class of considered parameters are topological parameters. And although the cross section shown in Fig. 1*A* is generated copying an experimentally determined hypocotyl cross section (determined using microscopy data) that has to be considered a naturally occurring topology, it remains open if the inherent asymmetries of this topology introduce a non-negligible bias into the model. We therefore tested the impact of these asymmetries and other features of this topology by comparing it to a range of altered topologies shown in Fig. 1*E* (including different cell size distributions).

In addition to shape and cell size distribution across layers (which at the same time influence cell volumes and thereby impact resulting auxin concentrations), the distribution of apoplast thicknesses across the different cell layers has to be considered as another important topological parameter because the thickness of the apoplastic space modifies auxin storage capabilities as well as possible auxin travel distances in the apoplast (again influencing apoplastic volumes and thereby impacting resulting auxin concentrations). To choose plausible thickness distributions for the simulations we relied on data gathered by Derbyshire and colleagues (Derbyshire et al. 2007). And in order to match the reported thickness distributions with the size of our seedlings, we used the growth stages IIa-c (Hayashi et al. 2010; Steinacher et al. 2012; Derbyshire et al. 2007). In addition to these reported distributions we tested different artificial and homogeneous apoplast thickness distributions of 100 nm, 400

nm, and 800 nm throughout all layers to avoid introducing unnecessary bias by potential peculiarities of the reported distributions.

In addition, auxin fluxes are subject to expression levels and localization of auxin carriers (Noh et al. 2003; Friml et al. 2002; Kerr & Bennett 2007; Yang et al. 2006). Here, especially the efflux carriers of the PIN family are of interest since they are known to show polar distributions as well as knock out experiments show a significant defect in phototropism (Friml et al. 2002; Ding et al. 2011; Christie et al. 2011). In addition to the PINs we explicitly incorporated potential contributions of carriers of the PGP family as well since they as well have been implicated to be of potential importance for phototropism (Noh et al. 2003; Christie et al. 2011; Nagashima et al. 2008). With respect to modeling auxin fluxes the other carriers and especially the carriers belonging to the AUX/LAX family were neglected due to their negligible effect on phototropism (Fig. S1).

Lastly, auxin fluxes are impacted by compartmental pH (Kramer & Bennett 2006; Kramer 2006; Krupinski & Jönsson 2010; Steinacher et al. 2012). Here, predominantly extracellular pH distributions are of interest because the natural occurring auxin indole-3-acetic acid (IAA) is a weak acid with a pK_a value of 4.8 (Delbarre et al. 1996). This means that for an assumed apoplast pH of around 5.5 (Kramer 2006; Krupinski & Jönsson 2010; Yu et al. 2000; Bibikova et al. 1998; Kurkdjian & Guern 1989), the protonation state of IAA crucially depends on the exact pH value (e.g. at pH 5.5 only ~16% of the IAA are protonated while at pH 4.8 already 50% are protonated, Fig. 1*C*). And considering that only protonated IAA is able to permeate cell membranes, the pH and protonation state potentially have a significant impact on auxin uptake by the cells. In this regard, we considered pH as an exogenous variable, i.e. one that follows an assumed distribution (e.g. homogeneous apoplastic

pH throughout the whole cross section during model equilibration and differential distributions as described during the simulations of gradient formation during photo stimulation). In other words, this means that the model does not consider inherent means on how photo stimulation impacts pH, e.g. by the hypothesized photo stimulus dependent regulation of H^+-ATPases or other yet uncovered means of regulation. Especially the hypothesis about a phototropin based regulation of H^+-ATPase provides a possible link between phototropism and apoplastic pH and could be included in a future version of the model inspired by the implementation by Steinacher and colleagues for a link between H^+-ATPase activity and pH .

Model equations

The model itself consists of ordinary differential equations modeling the auxin fluxes on the interfaces between the two considered types of compartments, cells and apoplast. This results in three different types of flux contributions: cellular auxin efflux, fluxes from the apoplast into adjacent cells, and fluxes between connected apoplast compartments. In turn these fluxes can be subsumed into one equation for each considered compartment type.

$$(1)\quad \frac{d[\mathrm{I}\quad_c]}{dt} = -\mu[\mathrm{I}\quad_c] + \sum_{i\in\mathcal{N}_\mathrm{a}(c)} \left(f_i^{\mathrm{I}}\quad[\mathrm{I}\quad_i] - f_c^{\mathrm{I}}\quad[\mathrm{I}\quad_c]\right) P_{\mathrm{diff}} A_{c,i}$$

$$- \sum_{i\in\mathcal{N}_\mathrm{a}(c)} \left(\frac{f_{cell}^{\mathrm{I}}\ {}^{-}[\mathrm{I}\quad_c]}{f_{cell}^{\mathrm{I}}\ {}^{-}[\mathrm{I}\quad_c] + K_{\mathrm{I}}}\right) C_{\mathrm{I}}\quad A_{c,i}$$

$$- \sum_{i\in\mathcal{N}_\mathrm{a}(c)} \left(\frac{f_c^{\mathrm{I}}\ {}^{-}[\mathrm{I}\quad_c]}{f_{cell}^{\mathrm{I}}\ {}^{-}[\mathrm{I}\quad_c] + K}\right) C\quad A_{c,i}$$

$$(2)\quad \frac{d[\mathrm{I}\quad _a]}{dt}$$

$$= \sum_{i\in\mathcal{N}_\mathrm{a}(a)} \frac{[\mathrm{I}\quad _i]-[\mathrm{I}\quad _a]}{d(m_a,m_i)} D_\mathrm{I} \quad \mathrm{A}_{a,i}$$

$$+ \sum_{i\in\mathcal{N}\ (a)} \left(f_i^\mathrm{I} \quad [\mathrm{I}\quad _i] - f_a^\mathrm{I} \quad [\mathrm{I}\quad _a]\right) P_\mathrm{diff} A_{a,i}$$

$$- \sum_{i\in\mathcal{N}\ (a)} \left(\frac{f_i^{\mathrm{I}\quad -}[\mathrm{I}\quad _i]}{f_i^{\mathrm{I}\quad -}[\mathrm{I}\quad _i] + K_\mathrm{I}}\right) C_\mathrm{I} \quad A_{a,i}$$

$$- \sum_{i\in\mathcal{N}\ (a)} \left(\frac{f_i^{\mathrm{I}\quad -}[\mathrm{I}\quad _i]}{f_i^{\mathrm{I}\quad -}[\mathrm{I}\quad _i] + K}\right) C \quad A_{a,i}$$

$$f_{\mathrm{om\,p}}^{\mathrm{I}} = \frac{1}{1 + 10^{\mathrm{p}\quad _{\mathrm{omp}} - pK_\mathrm{I}}} [\mathrm{I}\quad _{\mathrm{om\,p}}]$$

$$f_{\mathrm{om\,p}}^{\mathrm{I}\quad -} = \left(1 - f_{\mathrm{om\,p}}^{\mathrm{I}\quad -}\right)[\mathrm{I}\quad _{\mathrm{om\,p}}]$$

Here, $f_{\mathrm{om\,p}}^{\mathrm{I}}$ and $f_{\mathrm{om\,p}}^{\mathrm{I}\quad -}$ denote the protonated and deprotonated fractions of the total compartmental auxin concentration ($[\mathrm{I}\quad _{\mathrm{omp}}]$) in the compartment omp as a function of the dissociation constant of auxin (pK_I) and the compartmental pH (p $_{\mathrm{om\,p}}$). Then, eq. 1 represents the cellular auxin concentration changes over time, subject to proportional decay with decay rate μ, diffusive fluxes of protonated auxin between the considered cellular compartment c and all neighboring apoplast compartments $\mathcal{N}_\mathrm{a}(c)$, proportional to the respective contact surface $A_{c,i}$ and the membrane permeability for auxin (P_diff), and active efflux based on saturable transporters of the PIN or PGP family. The saturability of the active transport thereby

depends on the respective dissociation constants K_{I} and K and is subject to a transport capacity/density for the respective transporter combined with a constant reflecting membrane potential effects on active transport related fluxes (C_{I} or C) and the contact surface $A_{c,i}$ between cell c and apoplast compartment i. Conversely, the equation modeling the time evolution of auxin concentrations for apoplastic compartments ($[\mathrm{I}\quad_a]$) considers the same diffusive and active fluxes. Only the decay term is assumed to only act intracellularly but in addition to the already mentioned fluxes we assume free diffusion between neighboring apoplast compartments $\mathcal{N}_{\mathrm{a}}(a)$ guided by the concentration difference between compartments and relative to the distance $d(\cdot,\cdot)$ between centroids of the considered compartments (m_a and m_i) and proportional to the diffusion constant D_{I} and the contact surface $\mathrm{A}_{a,i}$.

Simulations

The model was implemented in MATLAB® version 2012b relying on the ode23tb numerical integrator which is an implementation of an explicit Runge-Kutta scheme (Bogacki & Shampine 1989). Simulation runs were conducted in a two step process; first a run for 2.5h of simulated time (here, timescales are set via the time-dependent diffusion and permeability rates) allowing the system to reach steady state under dark conditions (i.e. no polar PIN or heterogeneous pH distributions) before in another run of 4h simulated time the respective differential pH and PIN distributions are applied. Within these 4h of simulated time, all simulations reached a steady state, usually already after about 1.5h-2h. Here, this time window fits well auxin gradient formation timescales seen in root gravitropism (Band et al. 2012), which can be seen as a gradient formation process similar to the one underlying phototropism and therefore

indicating that at least the timescales in the simulations a well in line with naturally occurring events.

References

Band, L.R. et al., 2012. Root gravitropism is regulated by a transient lateral auxin gradient controlled by a tipping-point mechanism. *Proceedings of the National Academy of Sciences of the United States of America*, 109(12), pp.4668–4673.

Bibikova, T.N. et al., 1998. Localized changes in apoplastic and cytoplasmic pH are associated with root hair development in Arabidopsis thaliana. *Development (Cambridge, England)*, 125(15), pp.2925–2934.

Bogacki, P. & Shampine, L.F., 1989. A 3(2) pair of Runge-Kutta formulas. *Applied Mathematics Letters. An International Journal of Rapid Publication*, 2(4), pp.321–325.

Christie, J.M. et al., 2011. phot1 inhibition of ABCB19 primes lateral auxin fluxes in the shoot apex required for phototropism. *PLoS Biology*, 9(6), p.e1001076.

Delbarre, A. et al., 1996. Comparison of mechanisms controlling uptake and accumulation of 2,4-dichlorophenoxy acetic acid, naphthalene-1-acetic acid, and indole-3-acetic acid in suspension-cultured tobacco cells. *Planta*, 198(4), pp.532–541.

Derbyshire, P. et al., 2007. Cell elongation in Arabidopsis hypocotyls involves dynamic changes in cell wall thickness. *Journal of Experimental Botany*, 58(8), pp.2079–2089.

Ding, Z. et al., 2011. Light-mediated polarization of the PIN3 auxin transporter for the phototropic response in Arabidopsis. *Nature Cell Biology*, 13(4), pp.447–452.

Friml, J. et al., 2002. Lateral relocation of auxin efflux regulator PIN3 mediates tropism in Arabidopsis. *Nature*, 415(6873), pp.806–809.

Hayashi, Y. et al., 2010. Biochemical characterization of in vitro phosphorylation and dephosphorylation of the plasma membrane H+-ATPase. *Plant & cell physiology*, 51(7), pp.1186–1196.

Iino, M., 2001. Phototropism in higher plants. In D.-P. Häder & A. M. Breure, eds. *Comprehensive Series in Photosciences*. Amsterdam: Elsevier, pp. 659–811.

Kerr, I.D. & Bennett, M.J., 2007. New insight into the biochemical mechanisms regulating auxin transport in plants. *The Biochemical journal*, 401(3), pp.613–622.

Kramer, E.M., 2007. Computer models of auxin transport: a review and commentary. *Journal of Experimental Botany*, 59(1), pp.45–53.

Kramer, E.M., 2006. How Far Can a Molecule of Weak Acid Travel in the Apoplast or Xylem? *Plant Physiology*, 141(4), pp.1233–1236.

Kramer, E.M. & Bennett, M.J., 2006. Auxin transport: a field in flux. *Trends in plant science*, 11(8), pp.382–386.

Krupinski, P. & Jönsson, H., 2010. Modeling auxin-regulated development. *Cold Spring Harbor perspectives in biology*, 2(2), p.a001560.

Kurkdjian, A. & Guern, J., 1989. Intracellular pH: measurement and importance in cell activity. *Annual Review of Plant Physiology and Plant Molecular Biology*, 40(1), pp.271–303.

Lorrain, S. et al., 2009. Phytochrome interacting factors 4 and 5 redundantly limit seedling de-etiolation in continuous far-red light. *The Plant journal : for cell and molecular biology*, 60(3), pp.449–461.

Nagashima, A. et al., 2008. Phytochromes and cryptochromes regulate the differential growth of Arabidopsis hypocotyls in both a PGP19-dependent and a PGP19-independent manner. *The Plant journal : for cell and molecular biology*, 53(3), pp.516–529.

Noh, B. et al., 2003. Enhanced gravi- and phototropism in plant mdr mutants mislocalizing the auxin efflux protein PIN1. *Nature*, 423(6943), pp.999–1002.

Preuten, T. et al., 2013. Defining the site of light perception and initiation of phototropism in Arabidopsis. *Current Biology*, in press.

Steinacher, A., Leyser, O. & Clayton, R.H., 2012. A computational model of auxin and pH dynamics in a single plant cell. *Journal of theoretical biology*, 296, pp.84–94.

Tao, Y., et al., 2008. Rapid synthesis of auxin via a new tryptophan-dependent pathway is required for shade avoidance in plants. *Cell,* 133(1), pp.164-176.

Won, C., et al., 2011. Conversion of tryptophan to indole-3-acetic acid by TRYPTOPHAN AMINOTRANSFERASES OF ARABIDOPSIS and YUCCAs in Arabidopsis. *Proceedings of the National Academy of Sciences of the United States of America,* 108(45), pp.18518-18523.

Yang, Y.D. et al., 2006. High-affinity auxin transport by the AUX1 influx carrier protein. *Current Biology*, 16(11), pp.1123–1127.

Yu, Q., Tang, C. & Kuo, J., 2000. A critical review on methods to measure apoplastic pH in plants. *Plant and Soil*, 219(1), pp.29–40.

Supplemental figure legends

Supplementary Figure S1: Phototropic response of various *aux1lax* mutants and expression of *AUX1*, *LAX1*, *LAX2* and *LAX3* in etiolated hypocotyls.

(A) Time course of hypocotyl curvature upon unilateral blue light irradiation with a fluence rate of 10 µmol $m^{-2} s^{-1}$. Values are means +/- 2XSE, n>20. (B) Final bending angle of the indicated genotypes upon 24 hours of unilateral blue light irradiation with a fluence rate of 10 µmol $m^{-2} s^{-1}$. Values are means +/- 2XSE, n>40. (C) Relative expression of *AUX1*, *LAX1*, *LAX2* and *LAX3* in dissected etiolated hypocotyls. Data are averaged (+/- SD) from 3 biological replicates and expressed relative to one of the *AUX1* values.

Supplementary Figure S2: Impact of initial apoplastic pH on simulated auxin gradients. Decreasing the initial apoplastic pH from 5.5 to 4.8 overall results in approximately 50% weaker gradients. The impact on gradient formation is less in symmetric topologies, especially with smaller cells. This indicates that for a lowered base pH the cell size and thereby volume of cells might be of even greater importance than under higher resting pH of 5.5.

Supplementary Figure S3: Impact of different irradiation directions on simulated auxin gradients. To test the effect of topological features of our hypocotyl cross-section on auxin gradient formation we performed simulation following irradiation from different directions. Data are represented for the realistic cross section and an idealized cross section for the different apoplast configurations used in figure 2C.

Supplementary Figure S4: Phototropic response of *aha1-6* and *aha2-4* mutants

Data represent the hypocotyl curvature upon unilateral blue light irradiation with a fluence rate of 10 µmol m^{-2} s^{-1} for the indicated amount of time. Values are means +/- 2XSE, n>50.

Supplementary Figure S5: Hypocotyl elongation rate during phototropism

Average hypocotyl elongation rate during the first 4 hours of unilateral blue light irradiation (fluence rate of 10 µmol m^{-2} s^{-1}) in Wild Type, *aha1-6* and *aha2-4* mutants (A); upon DCCD treatment (B); or upon FC treatment (C). Values are means +/- 2XSE, n > 20.

Supplementary Figure S6: Analysis of auxin distribution in hypocotyls

(A) Method used for quantification. (B) DII-Venus signal before (left) and after (right) unilateral blue light irradiation (fluence rate of 10 µmol m^{-2} s^{-1}), in seedlings treated with DMSO, NPA and FC. (C) Kinetics of phototropism in conditions used for DII-Venus signal detection. Data represent the rate of hypocotyl growth curvature upon unilateral blue light irradiation with a fluence rate of 10 µmol m^{-2} s^{-1}, for wild type seedlings grown for 4 days in darkness (4d etiolated), 3 days in darkness and 24 hours in white light (25 µmol m^{-2} s^{-1}) and treated with DMSO, NPA of FC. For comparison, the data obtained using 3 day-old etiolated WT seedlings (figS3) are indicated (dashed line). Values are means +/- 2XSE, n>20.

Supplementary Figure S7: Quantification of H+-ATPase phosphorylation levels at the penultimate Thr. Western blots used to generate the quantitative data presented on Figure 6 (a. u. arbitrary units).

Supplementary Figure S8: Phototropic response of the auxin biosynthesis mutants *sav3* and *yuc1yuc4*. 3-day-old, etiolated seedlings grown on vertical plates with half-strength MS agar medium were irradiated with unilateral blue light at a fluence rate of 1 µmol m^{-2} s^{-1}. Curvature was determined at the indicated times after the start of irradiation. As both mutants are in the Col-0 background, bending rates were compared to Col-0 wild-type seedlings. Data shown are means +/- two standard errors of 136 (WT), 146 (*yuc1yuc4*), and 112 (*sav3*) measured seedlings.

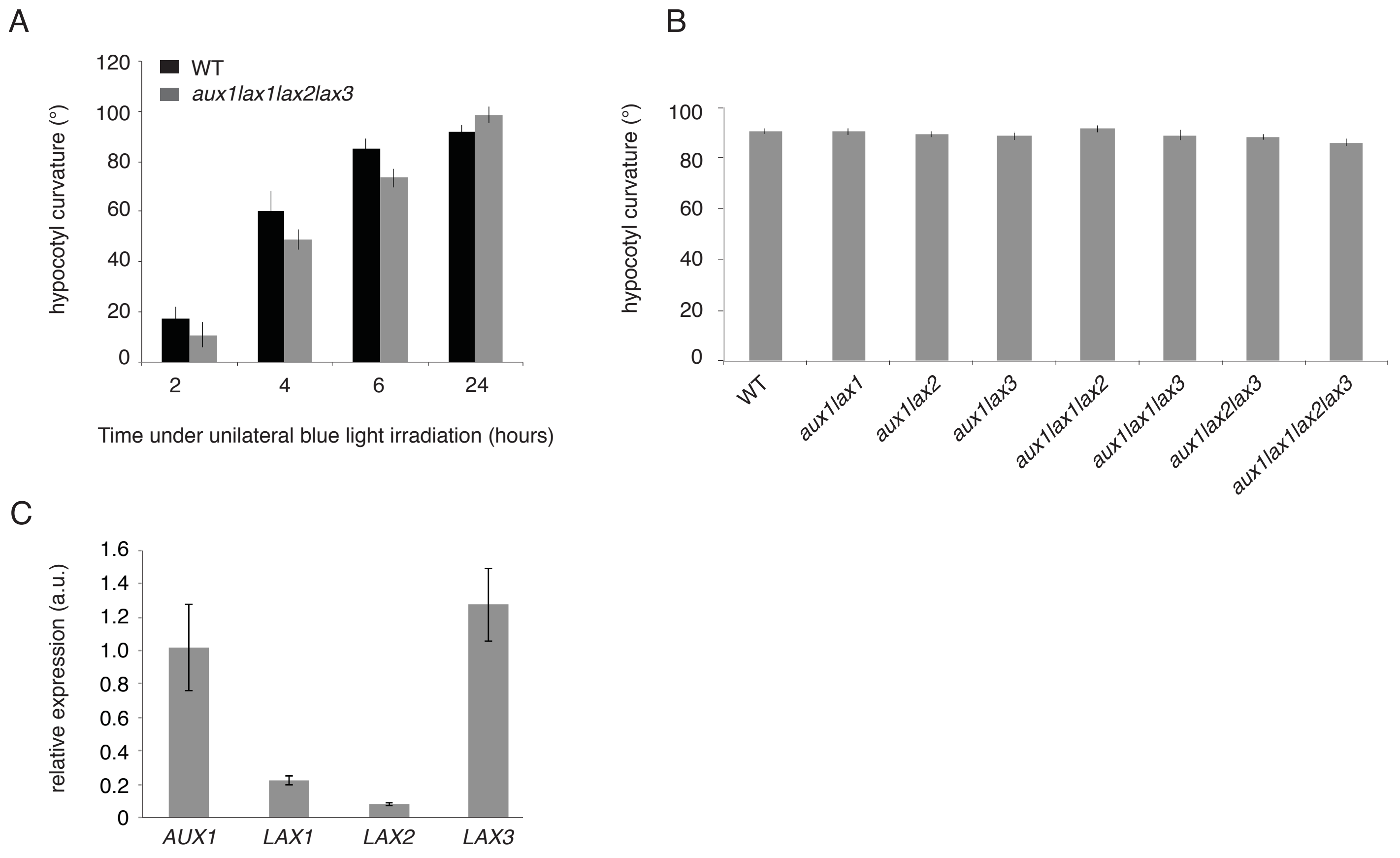

Supplementary Figure S1

Gradient formation results comparison between resting pH 5.5 and resting pH 4.8

based on scenario with concomittant epidermal pH change

Apoplast thickness, cell group 1:

resting pH	PIN activity	IIa (before elongation & thick)	IIb (medium elongation & medium thickness)	IIc (elongated & thin)	100nm (homogeneous & thin)	400nm (homogeneous & medium)	800nm (homogeneous & thick)
pH 5.5	normal PIN activity	6.7	4.4	4.3	3.4	5.3	6.5
	reduced PIN activity	5.1	3.2	3.1	1.1	4.0	5.0
pH 4.8	normal PIN activity	1.6	0.6	0.6	0.4	0.9	1.5
	reduced PIN activity	1.3	0.5	0.4	0.3	0.7	1.2

Apoplast thickness, cell group 2:

resting pH	PIN activity	IIa (before elongation & thick)	IIb (medium elongation & medium thickness)	IIc (elongated & thin)	100nm (homogeneous & thin)	400nm (homogeneous & medium)	800nm (homogeneous & thick)
pH 5.5	normal PIN activity	7.4	5.4	5.2	4.8	6.9	7.9
	reduced PIN activity	5.8	4.0	3.9	3.5	5.2	6.2
pH 4.8	normal PIN activity	3.7	0.6	0.5	1.8	3.1	3.8
	reduced PIN activity	2.8	0.4	0.4	1.3	2.3	3.0

Apoplast thickness, cell group 3:

resting pH	PIN activity	IIa (before elongation & thick)	IIb (medium elongation & medium thickness)	IIc (elongated & thin)	100nm (homogeneous & thin)	400nm (homogeneous & medium)	800nm (homogeneous & thick)
pH 5.5	normal PIN activity	2.7	2.1	2.0	1.6	2.1	2.5
	reduced PIN activity	2.0	1.6	1.5	1.2	1.6	1.9
pH 4.8	normal PIN activity	1.5	1.0	0.9	0.8	1.1	1.3
	reduced PIN activity	1.1	0.7	0.7	0.6	0.8	1.0

Apoplast thickness, cell group 4:

resting pH	PIN activity	IIa (before elongation & thick)	IIb (medium elongation & medium thickness)	IIc (elongated & thin)	100nm (homogeneous & thin)	400nm (homogeneous & medium)	800nm (homogeneous & thick)
pH 5.5	normal PIN activity	6.1	5.3	5.3	5.1	5.9	6.4
	reduced PIN activity	4.8	4.0	3.9	3.7	4.4	5.0
pH 4.8	normal PIN activity	3.8	3.0	3.0	2.8	3.5	4.0
	reduced PIN activity	2.9	2.2	2.2	2.0	2.6	3.1

Apoplast thickness, cell group 5:

resting pH	PIN activity	IIa (before elongation & thick)	IIb (medium elongation & medium thickness)	IIc (elongated & thin)	100nm (homogeneous & thin)	400nm (homogeneous & medium)	800nm (homogeneous & thick)
pH 5.5	normal PIN activity	2.1	1.5	1.5	1.4	1.9	2.2
	reduced PIN activity	1.6	1.1	1.1	1.0	1.4	1.9
pH 4.8	normal PIN activity	1.2	0.8	0.7	0.6	1.0	1.2
	reduced PIN activity	0.9	0.7	0.5	0.5	0.7	0.9

cell size and distribution

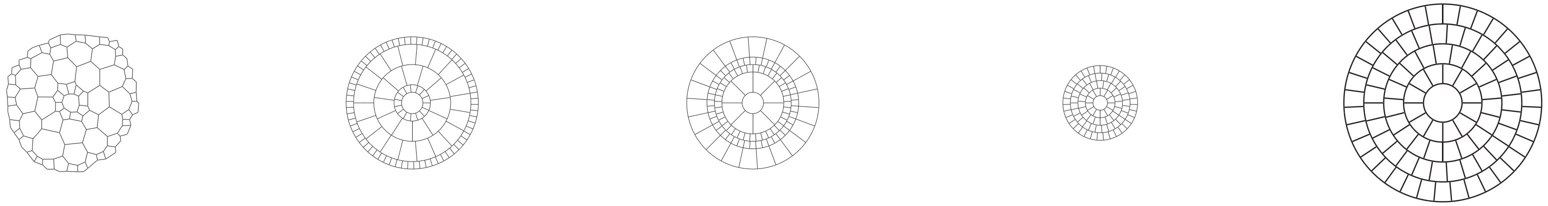

concentration difference in percent (%)

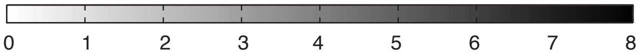

Supplementary Figure S2

Gradient formation results comparison between different directions of light stimulus

based on scenario with concomittant epidermal pH change at resting pH 5.5

direction of incoming light	IIa (before elongation & thick)	IIb (medium elongation & medium thickness)	IIc (elongated & thin)	100nm (homogeneous & thin)	400nm (homogeneous & medium)	800nm (homogeneous & thick)	IIa (before elongation & thick)	IIb (medium elongation & medium thickness)	IIc (elongated & thin)	100nm (homogeneous & thin)	400nm (homogeneous & medium)	800nm (homogeneous & thick)
direction 1	6.7	4.3	4.3	3.4	5.3	6.5	7.4	5.4	5.2	4.8	6.9	7.9
direction 2	7.4	4.6	4.6	3.4	5.8	7.2	7.4	5.3	5.2	5.1	7.2	8.0

apoplast thickness

topology

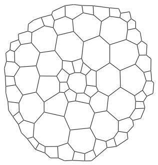

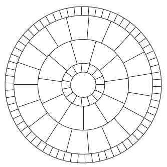

concentration difference in percent (%)

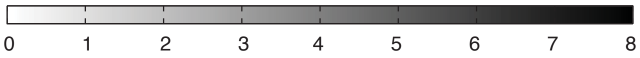

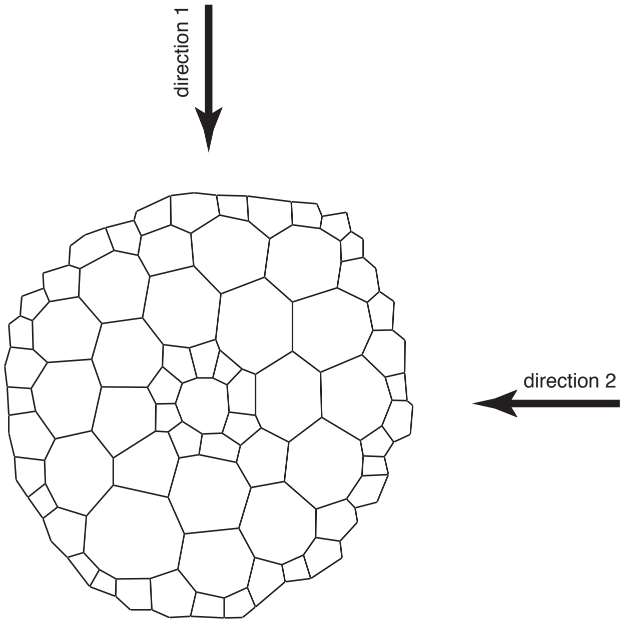

Supplementary Figure S3

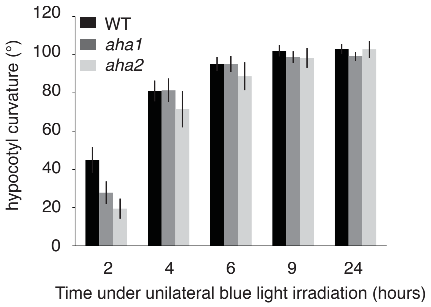

Supplementary Figure S4

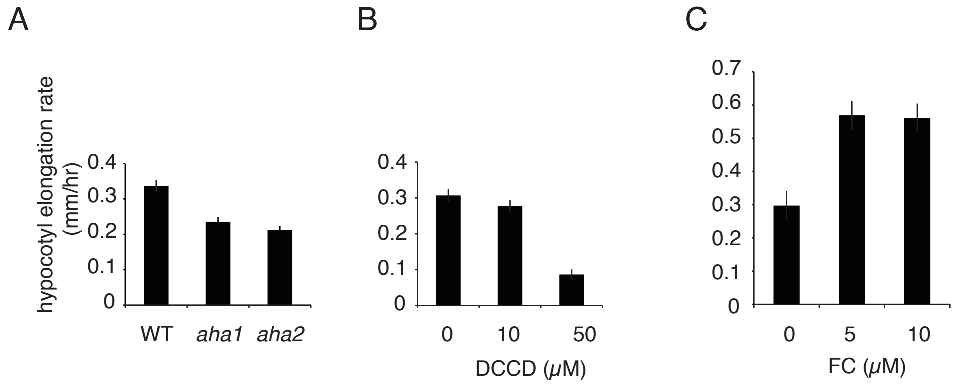

Supplementary Figure S5

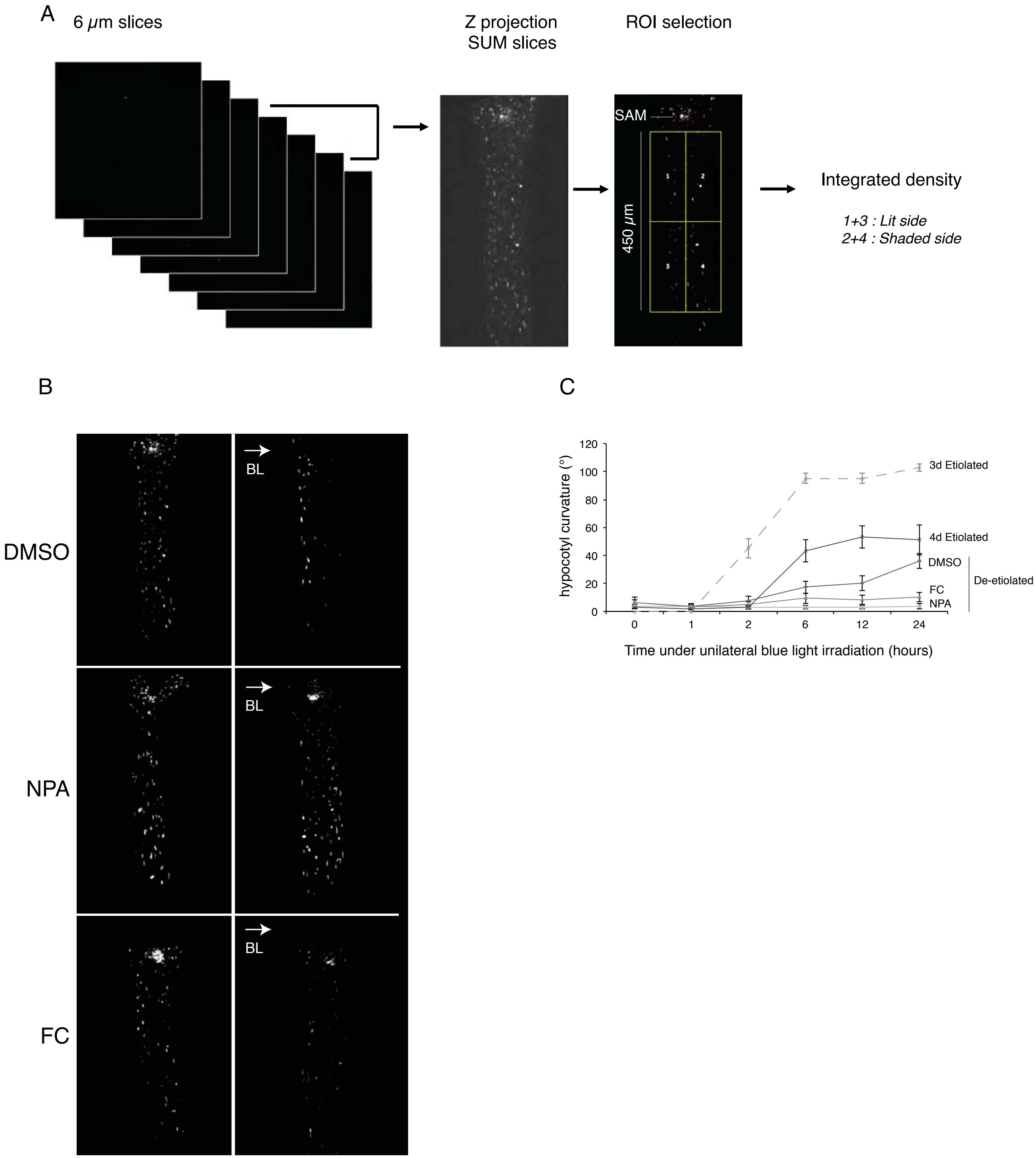

Supplementary Figure S6

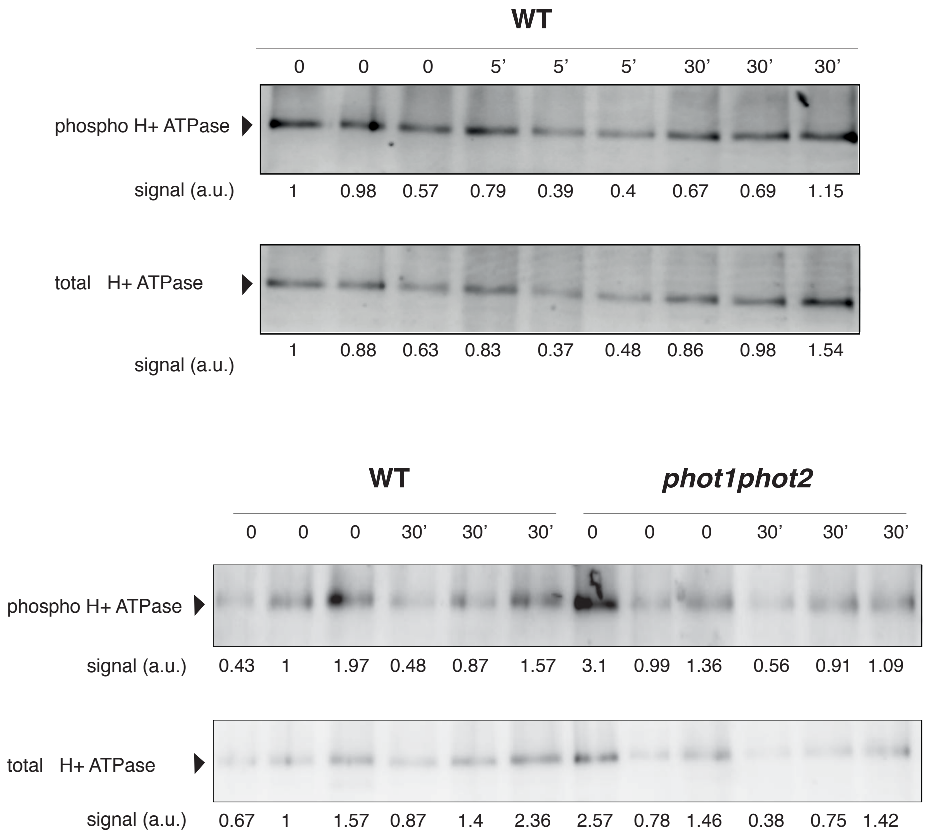

Supplementary Figure S7

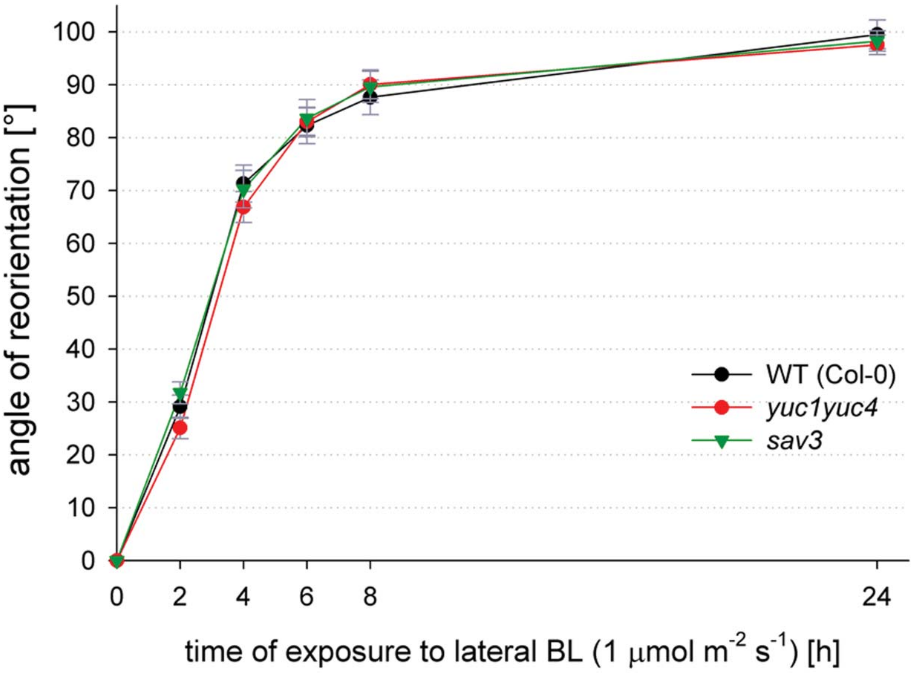

Supplementary Figure S8

Supplementary Table S1: Parameters impacting auxin flux kinetics including their assumed values. In the model, capacities are subject to a base pumping rate combined with membrane potential effects (last factor, expressed as number of particles per time unit and per area unit of cell surface) and a loading density of membranes with the respective carrier (PGP or PIN). Because PIN carriers are assumed to potentially be polarly distributed, we assumed different base loadings (factors in the bracket) as carrier densities.

Parameter	Function	Value
D	Auxin diffusion constant	0.0024 cm^2 h^{-1} (1)
P	Auxin membrane permeability	0.2 cm h^{-1} (1)
**μ**	Auxin decay rate	0.00075 s^{-1}
K	Dissociation constant of PINs and auxin	30
C	Auxin transport capacity for PINs	{0.03, 0.07, 0.15, 0.65} * 4.7 molecules/surface unit
K	Dissociation constant of PGPs and auxin	30
C	Auxin transport capacity for PGPs	0.5 * 4.7 molecules/surface unit

(1) Kramer EM (2006) How far can a molecule of weak acid travel in the apoplast or xylem? Plant Physiol 141(4):1233-1236.

Supplementary Table S2: List of primers used in this study

Gene		name	sequence	efficiency	r2
**AUX1**	for	LAP009	CGGAAACCGTACCGGGAAAG		
	rev	LAP010	GAACCAAGCGTCCCAGACAG	2	0.999
**LAX1**	for	LAP015	CCCTCCCTCTTCCATTAAATC		
	rev	LAP016	TTTCTCACCCGACATTAGC	1.93	0.996
**LAX2**	for	LAP025	AGATGGAGAACGGTGAGAAAGC		
	rev	LAP026	TGAGAACGAGTATGGCAGTGTC	1.994	0.993
**LAX3**	for	LAP029	CGCAGGGAATTACTTAGAAATG		
	rev	LAP030	TGTGAGAACGAGTATGGTAATG	1.98	0.992
